# PGE2 promotes breast cancer-associated lymphangiogenesis by activation of EP4 receptor on lymphatic endothelial cells

**DOI:** 10.1186/s12885-016-3018-2

**Published:** 2017-01-05

**Authors:** Pinki Nandi, Gannareddy V. Girish, Mousumi Majumder, Xiping Xin, Elena Tutunea-Fatan, Peeyush K. Lala

**Affiliations:** 1 Department of Anatomy and Cell Biology, University of Western Ontario, London, Ontario N6A5C1 Canada; 2 Department of Oncology, University of Western Ontario, London, Ontario N6A5C1 Canada; 3 Children’s Health Research Institute, Schulich School of Medicine and Dentistry, University of Western Ontario, London, Ontario N6A5C1 Canada; 4 Department of Biology, Brandon University, Brandon, Manitoba R7A 6A9 Canada

**Keywords:** PGE2, Cyclooxygenase (COX)-2, Lymphangiogenesis, Angiognesis, EP4 receptors, EP4 antagonist, Breast cancer, Metastasis, Lymphatic endothelial cells, Directed in vivo lymphangiogenesis assay (DIVLA)

## Abstract

**Background:**

Lymphatic metastasis, facilitated by lymphangiogenesis is a common occurrence in breast cancer, the molecular mechanisms remaining incompletely understood. We had earlier shown that cyclooxygenase (COX)-2 expression by human or murine breast cancer cells promoted lymphangiogenesis and lymphatic metastasis by upregulating VEGF-C/D production by tumor cells or tumor-associated macrophages primarily due to activation of the prostaglandin receptor EP4 by endogenous PGE2. It is not clear whether tumor or host-derived PGE2 has any direct effect on lymphangiogenesis, and if so, whether EP4 receptors on lymphatic endothelial cells (LEC) play any role.

**Methods:**

Here, we address these questions employing in vitro studies with a COX-2-expressing and VEGF-C/D-producing murine breast cancer cell line C3L5 and a rat mesenteric (RM) LEC line and in vivo studies in nude mice.

**Results:**

RMLEC responded to PGE2, an EP4 agonist PGE1OH, or C3L5 cell-conditioned media (C3L5-CM) by increased proliferation, migration and accelerated tube formation on growth factor reduced Matrigel. Native tube formation by RMLEC on Matrigel was abrogated in the presence of a selective COX-2 inhibitor or an EP4 antagonist. Addition of PGE2 or EP4 agonist, or C3L5-CM individually in the presence of COX-2 inhibitor, or EP4 antagonist, restored tube formation, reinforcing the role of EP4 on RMLEC in tubulogenesis. These results were partially duplicated with a human dermal LEC (HMVEC-dLyAd) and a COX-2 expressing human breast cancer cell line MDA-MB-231. Knocking down EP4 with shRNA in RMLEC abrogated their tube forming capacity on Matrigel in the absence or presence of PGE2, EP4 agonist, or C3L5-CM. RMLEC tubulogenesis following EP4 activation by agonist treatment was dependent on PI3K/Akt and Erk signaling pathways and VEGFR-3 stimulation. Finally in a directed in vivo lymphangiogenesis assay (DIVLA) we demonstrated the lymphangiogenic as well as angiogenic capacity of PGE2 and EP4 agonist in vivo.

**Discussion/conclusions:**

These results demonstrate the roles of tumor as well as host-derived PGE2 in inducing lymphangiogenesis, at least in part, by activating EP4 and VEGFR-3 on LEC. EP4 being a common target on both tumor and host cells contributing to tumor-associated lymphangiogenesis reaffirms the therapeutic value of EP4 antagonists in the intervention of lymphatic metastasis in breast cancer.

## Background

Lymphatic metastasis is a frequent occurrence in most epithelial cancers [1], including cancers of the breast [2], stomach [3], colon [4], pharynx and larynx [5], lungs [6], uterine cervix [7], prostate [8] and the ovary [9], negatively impacting patient survival. This is also the first route of spread for many cancers, which can subsequently metastasize from the lymph nodes to other organs via the blood stream. A positive association between intra-tumoral and /or peri-tumoral lymphangiogenesis with lymphatic metastasis noted in many cancers e.g., cancers of the pharynx and larynx [5] and the breast [10, 11], suggests that newly formed lymphatics serve as conduits for entry and spread of cancer cells to lymph nodes. Development of new lymphatic capillaries (lymphangiogenesis) is an event common to inflammation and carcinogenesis suggesting a commonality of molecular players in both events. Numerous ligands such as VEGF-C, VEGF–D, neuropilins, and certain chemokines have been shown to exert a direct growth-stimulatory effect on cancer-associated lymphatic endothelial cells (LEC) by activation of their respective receptors [12–15]. However, the roles of prostaglandin (PG) E2, another key inflammation-associated molecule, remain to be explored fully in breast cancer-associated lymphatic outgrowth.

Expression of cyclooxygenase (COX)-2, an inflammation-associated enzyme stimulates progression and metastasis of a variety of cancers including breast cancer [16–18]. COX-2 promotes lymphatic metastasis of postpartum breast cancer [19]. PGE2 in the tumor micro-environment resulting from elevated COX-2 expression by cancer or host cells promotes breast cancer progression by multiple mechanisms: inactivation of host anti-tumor immune cells [20, 21], stimulation of tumor cell migration [22, 23], invasiveness [23, 24], induction of stem-like cells (SLC) [25], tumor-associated angiogenesis [22] and lymphangiogenesis [26]. The latter results from an upregulation of lymphangiogenic growth factors VEGF-C [26] or VEGF-D in tumor cells [27] or recruitment of VEGF-C/D producing macrophages at the tumor site [25]. COX-2 mediated promotion of migratory [23], invasive [24], SLC stimulatory [25] and VEGF-C and –D up-regulatory [25–27] functions were primarily due to activation of the PGE receptor EP4 expressed by breast cancer cells [26, 27] as well as tumor infiltrating macrophages [25], suggesting that both COX-2 and EP4 are good therapeutic targets for abrogating all these PGE2 mediated functions. We validated this contention in a high COX-2 expressing syngeneic murine breast cancer model, in which therapy with a COX-2 inhibitor or two EP4 antagonists, equally inhibited tumor growth, tumor-associated angiogenesis and lymphangiogenesis and metastasis to the lymph nodes and the lungs [27], and were also SLC-reductive in vivo [25]. Thus EP4 was identified as a common target on cancer cells and macrophages to abrogate multiple events: angiogenesis, lymphangiogenesis, metastasis, and cancer stem cell phenotype. It was, however, unclear whether tumor or host derived PGE-2 had any direct effect on lymphatic endothelial cells, and if so, whether EP4 receptors on LECs played any role.

To answer these questions, we utilized lymphangiogenesis assays conducted in vitro and in vivo. In vitro assays employed rat mesenteric lymphatic endothelial cells (RMLEC), and also human dermal-derived LEC (HMVEC-dLyAd) in some experiments. Proliferation, migration and capillary-like tube formation by the LEC plated on growth factor- reduced (GFR) Matrigel was quantified under various experimental conditions including genetic manipulation of the LEC or exposing LEC to cell-free conditioned media from COX-2 high murine or human breast cancer cell lines. We addressed whether: (a) the native tube forming capacity of the LEC on Matrigel is dependent on COX-2 or EP4 activity; (b) soluble products of COX-2-expressing breast cancer cells stimulate tube formation by the LEC; (c) proliferation, migration and tube forming capacity of the LEC are stimulated by exogenous PGE2 or selective EP4 agonists and if so, what are the underlying signaling mechanisms. In additional experiments we utilized a directed *in vivo* lymphangiogenesis assay (DIVLA) devised in our laboratory [28, 29] to examine the roles of exogenous PGE2 and EP4 agonists in promoting lymphatic vessel outgrowth in nude mice. Results revealed that tumor or host-derived PGE2 in the tumor micro-environment or exogenous PGE2 or EP4 agonists can directly stimulate lymphangiogenesis by activation of EP4 receptors on the LEC via PI3K/Akt and Erk signaling pathways and VEGFR-3 stimulation, so that EP4 antagonists may be useful in the prevention and intervention of lymphatic metastasis in breast cancer.

## Methods

### Reagents

DMEM-F12 medium, Fetal bovine serum (FBS), Dulbecco’s phosphate buffered saline (DPBS), trypsin, glutamine, sodium pyruvate, and nonessential amino acids, 0.25% Trypsin-EDTA and Penicillin/Streptomycin used in cell culture were obtained from Gibco, Life technologies (Burlington, ON). BD Falcon cell culture flasks (75cm^2^), 6-well plates, 24-well

plates, growth factor reduced (GFR) Matrigel were from BD Biosciences, San Jose, CA, USA. Antibodies raised against VEGF-C (SC-1881), VEGF-D (SC-6314), β-actin (SC-47778), CD-31 (SC-376764), Lyve-1 (SC-80170), COX-2 (SC-1747) and rat EP4 shRNA (sc-270389-SH) were from Santa Cruz Biotechnology, Santa Cruz, CA. Prox-1 (11–002) antibody were from Angiobio, Del Mar, CA, USA. EP4 antibody (101775), PGE2, PGE2 ELISA kit and, PGE1OH, L902 688 (both EP4 agonist) and NS-398 (selective COX-2 inhibitor) and were from Cayman, Ann Arbor, MI, USA. M-PER® Mammalian Protein Extraction Reagent, HALT™ Protease Inhibitor Cocktail and Restore™ Plus Western blot stripping buffer were from Pierce, Rockford, IL, USA. Goat anti-rabbit IgG and goat anti-mouse IgG linked HRP secondary antibodies were from Bio-Rad, Hercules, CA. qRT-PCR primers were designed using Primer-3 site and synthesized at the UWO Oligo factory. RNeasy Mini Kit was from Qiagen, qScript™, cDNA Synthesis Kit and PerfeCTa® Green SuperMix from Quanta Biosciences, Gaithersberg, MD, USA; Indomethacin (non-selective COX-1/COX-2 inhibitor) from Sigma (Oakville, ON, Canada) and selective EP4 antagonist RQ15986 was a gift from RaQualia Pharma Inc (Ask/At), Japan. Sources of other reagents are given in parenthesis: Isoflurane (Baxter, ON, Canada), rabbit anti-mouse Lyve-1 antibody (Cat No 11–034, AngioBio, USA), Alexa Fluor 594 (Invitrogen, CA) anti rabbit secondary antibody, rat monoclonal CD31 antibody (MEC 13.3, Santa Cruz Biotechnology), Alexa Fluor 594 Goat Anti-Rat IgG (H + L), Alexa Fluor 647 Donkey Anti-Rabbit IgG (H + L) secondary antibodies, Vectashield solution (Vector Laboratories, Burlington, ON). Cultrex® DIVAA Starter Kit, CellSperse solution (Cat# 3450-048-05), wash buffer (Cat# 3450-048-03), DIVAA™ 1X Dilution Buffer (Cat# 3450-048-07) were from Trevigen, MD, USA. O.C.T. compound (Tissue-Tek*, Sakura Finetek USA, Inc., Torrance, USA). Taqman primers for murine LYVE-1 (Mm00475056_m1), CD31 (Mm01242584_m1) and β-actin (4352933E) with TaqMan Gene Expression Assays kit was from Applied Biosystems, USA. Endothelial Cell Growth Medium EGM-2-MV Bulletkit (CC-3202) was from Lonza, MO. USA.

### Mice

Six weeks old Athymic nude female mice (Hsd: Athymic Nude-*Foxn1* ^*nu*^ */Foxn1* ^*+*^), were obtained from Harlan laboratories, Indianapolis, IN and used at 8 weeks of age.

### Cell lines and culture

Rat mesenteric lymphatic endothelial cell line (RMLEC) is a spontaneously immortalized LEC isolated from rat mesenteric lymphatic endothelium [30], kindly provided by Dr. Sophia Ran, Southern Illinois University School of medicine. RMLEC (at passages 40–45) was grown in DMEM supplemented with 10% FBS, 2mM glutamine, 50 U/ml penicillin and 50μg/ml streptomycin, 1mM Sodium pyruvate, and 1mM nonessential amino acids at 37 °C and 10% CO_2_ [30]. RMLECs exhibited strong levels of expression of lymphatic endothelium specific markers, including Prox-1, and Lyve-1. C3L5 is a COX-2 expressing and highly metastatic murine breast cancer cell line, clonally derived from a spontaneous mammary adenocarcinoma in C3H/HeJ mice in our laboratory and is not commercially available [31]. C3L5 cells were grown in advanced DMEM medium without serum for 24h to collect conditioned medium (CM) to study the functional effects of the CM on the RMLEC. Human Dermal Lymphatic Microvascular Endothelial Cells (HMVEC-dLyAd) (Cat. # CC-2810T25) obained from Clonetics/Lonza (Walkersville, MO) and maintained in an endothelial growth medium were used at passage 5. Human breast cancer MDA-MB-231 cell line (Cat. # ATCC® HTB­ 26™) (used at passage 3) obtained from the ATCC and were grown in RPMI 1640 medium (Invitrogen, Burlington, ON) supplemented with 10% FBS, 100 U/ml penicillin, and 100μg/ml Streptomycin. MDA-MB-231 cell CM was collected as above, to examine functional effects on HMVEC-dLyAd cells.

### LEC proliferation assay

Serum-starved RMLEC cells were seeded onto 96-well tissue-culture microplates, treated with either PGE2 (10μM), PGE1OH (10μM) or C3L5 CM for 24 h, and a cell proliferation ELISA BrdU (colorimetric) assay (Roche Applied Science, Indianapolis, IN, USA) was performed.

### LEC migration assay

Migration of RMLEC (wild-type or EP4 silenced) was assessed with Boyden chambers using Transwell® inserts (Corning Life Sciences, Oneonta, NY, USA) separated by a polycarbonate membrane with 8 μm pore opening placed within 24-well plates. A 300μl suspension of serum-starved RMLEC cells at a concentration of 2 × 10^5^/ml of serum free medium were seeded in the upper chamber in the presence of either PGE2, PGE1OH or C3L5- CM while lower chamber had 2% charcoal-dextran stripped FBS containing medium (free of PGE2). The assembled chambers were then incubated for 24 h. After incubation, the cells from the top of the membrane were wiped off with cotton swabs whereas the migrant cells (from the bottom of the membrane) were fixed with cold methanol, stained with eosin/thiazine, and washed with distilled water. The membranes were then dried, cut with surgical blade, and fixed with mounting medium on a glass slide. Direct microscopic counting at 40 × magnification (Leica DFC 295, Leica Microsystems, Germany) of cells that have migrated to the lower side of the membrane was performed and a mean value for each sample was calculated.

### LEC tube formation assay

This assay was carried out with wild-type or EP4 silenced RMLEC and native HMVEC-dLyAd cells under different treatment conditions (as specified later in results) on GFR Matrigel. Matrigel was thawed overnight at 4 °C, diluted with cold sterile PBS in 1:1 ratio, and used to coat 24-well culture plates (0.25 ml/well) and left at 37 °C for 6 h. After polymerization, 40,000–60,000 cells/well, suspended in Advanced DMEM medium (Invitrogen) were added to each well. Under native serum-free conditions very low levels of tube formation occurred with wild-type RMLEC at 12 h, gradually increasing with time (peaking between 24 and 30 h). The tempo of native tube formation in HMVEC-dLyAd cells was somewhat slower peaking at 48 h. Under conditions of stimulation tube formation in both native LECs was enhanced and sustained for 96 h. Pictures of 10–15 random fields were captured in various experiments using a Leica Microscope EC3 camera. The numbers of branching points or total tube lengths per unit area were quantified using the NIH ImageJ software. Branching points were considered as points from which two or more tubes branched, as reported earlier [28]. This method gave similar answers obtained by measurements of total tube lengths per unit area [15]. Both methods were used in the current study.

### Western blot for Lyve-1, Prox-1, CD-31, COX-2, EP4, VEGF-C, VEGF-D

RMLEC cells were washed with ice-cold DPBS (including 10nM NaF and 1mM Na_3_VO_4_) and lysed in M-PER® lysis buffer supplemented with HALT™ protease inhibitor cocktail, 10mM NaF and 1mM Na_3_VO_4_. Cell lysates were centrifuged and supernatant protein was quantified using the BCA protein assay kit. Equal amounts of protein (25μg) were separated on 12% SDS-PAGE gels and transferred to a PVDF membrane. Membranes were blocked in 5% non-fat milk in TBS (20mM tris-base, 0.14M NaCl, pH 7.8) with 0.05% Tween-20 for one hour at room temperature and probed for respective primary antibody (1:1000) at 4 °C, overnight. The membranes were washed with TBS with 0.05% Tween-20 and incubated in HRP-conjugated rabbit or mouse secondary antibodies (1:5000) for one hour at room temperature. Peroxidase activity was detected with enhanced chemiluminescence reagent.

### Real time quantitative PCR for COX-2, EP4, VEGF-C, VEGF-D

Total RNA was extracted from RMLECs after specific treatments as detailed in the results using RNeasy Mini Kit. Respective cDNA was synthesised using qScript™ cDNA Synthesis Kit, and real time quantitative PCR analysis was performed with BioRad thermocyler using PerfeCTa® Green SuperMix and data analysed using CFX Manager™ software for *Rattus norvegicus* β-actin, VEGF-C, VEGF-D, COX2, EP4 gene expression. To determine the relative levels of gene expression, the comparative threshold cycle method (ΔCt) was use [32]. The final mRNA levels were normalized according to their Ct values from the standard curves and expressed in relation to respective *β*-actin/GAPDH level. The following primer pairs have been used: β-actin forward (5-tag gtt ttg tca aag aaa gg-3), reverse (5-tag gtt ttg tca aag aaa gg-3); COX-2 forward (5-tac tac gcc tga gtt tct ga-3) reverse (5-ggt gta gta gga gag gtt gg-3); VEGF-C forward (5-ctt gaa aaa ctg ttg cca ca-3) reverse (5-aca aga gaa aaa cct cag ct-3); VEGF-D forward (5-tat gaa cac aag cac ctc ct-3) reverse (5-gac att gat ctt ctt ctg gg-3); EP4 forward (5-ctg tgc tca gta aag cca ta-3) reverse (5-ctt tca gtt agg tct ggc ag-3); GAPDH:forward (5’-tgattctacccacggcaagtt-3’) reverse (5’-tgatgggtttcccattgatga-3’). The formed respective amplicons were verified by running a DNA agarose gel along with a standard DNA marker.

### Knock down of EP4 gene in RMLEC

Rat EP4 shRNA plasmid (sc-270389-SH) and control shRNA (sc-108066) plasmid were transfected stably into RMLEC cells using an AMAXA machine and Amaxa Cell Line Nucleofector Kit V (cat # VCA-1003) and protocol. After transfection, cells were selected in RMLEC medium with 10μg/ml puromycin and maintained with 300ng/ml puromycin. The knockdown was validated with a qPCR, showing 80% downregulation in EP4-Knock down (KD) cells.

### ELISA

ELISA was performed for PGE2 with RMLEC cell lysates following the procedure specified in the PGE2 ELISA kits. In brief RMLECs grown to reach 60% confluence under specific treatment conditions were washed with PBS and incubated in SFM for 12h. Cells were washed with PBS twice and incubated with trypsin and the cell suspension were centrifuged and re-suspended in the media and the cell number was calculated using a cell counter. Cells were re-centrifuged to obtain the cell pellet which was lysed with RIPA lysis buffer and protein extracted. The protein extract for each of the conditions in triplicate was incubated in the ELISA plates and ELISA performed for PGE2 with respective standards supplied along with the kit. The experiment was performed in triplicate in two separate experiments and the concentrations of the PGE2 were extrapolated from the standard curve.

### Directed In-vivo lymphangiogenesis assay (DIVLA)

This assay was conducted as reported by us [29]. In brief 6 weeks old athymic nude female mice were allowed to acclimatize for 2 weeks, maintained on standard mouse chow and tap water on a 12 h light/dark cycle. Sterile angioreactors were pre-chilled at 4° C and filled with 20μl of either growth factor reduced Basement Membrane Extract (BME) (Cat# 3450-048-02, Trevigen, MD, USA) alone, or in combination with PGE2 (10μM) or EP4 agonists PGE1OH and L 902688 (10uM) or VEGF-C (20ng) (2179-VC-025, R&D Systems, CA), the latter serving as positive control for lymphangiogenesis and angiogenesis. Angioreactors were incubated at 37 °C for 1 h to allow BME gel formation, before subcutaneous implantation into the mice. Mice were anaesthetized with isoflurane. Four angioreactors containing the same agents were implanted per mouse, two on each side of the dorsal flank region, and 2 mice were used per condition. After a period of 10 days, mice were euthanized and the angioreactors were retrieved and used for three purposes as reported earlier [29]. All the angioreactors were retrieved without severing the ingrowing vessels and excising the rest of the tissues with fine scissors. One angioreactor was flash-frozen immediately with dry ice for making cryosections. The other three were used to collect cellular contents and conduct lymphatic in-growth assay and RNA extracted for real time gene expression. Three approaches were utilized for measuring angiogenesis/lymphangiogenesis: (i) Lyve-1, prox-1 and CD31 proteins by immunoflurometry of cell lysates; (ii) Lyve-1, prox-1 and CD31 mRNA by qPCR of extracted RNA; (iii) a visual quantification of lymphatic and blood vessel in-growth into the angioreactors from double labeling for Lyve-1, or Prox-1, (LEC markers) and CD31 (blood vessel endothlial marker) using the hot spot method [25, 27].

### Statistical analysis

Data are presented as mean ± standard error (SE) for each treatment compared to the control. Graph-pad Prism5 software were used to analyze the data with *t*-test. Differences between two treatment groups were accepted as significant at *p* < 0.05.

## Results

### Expression of LEC markers by RMLEC

**Fig. 1:**
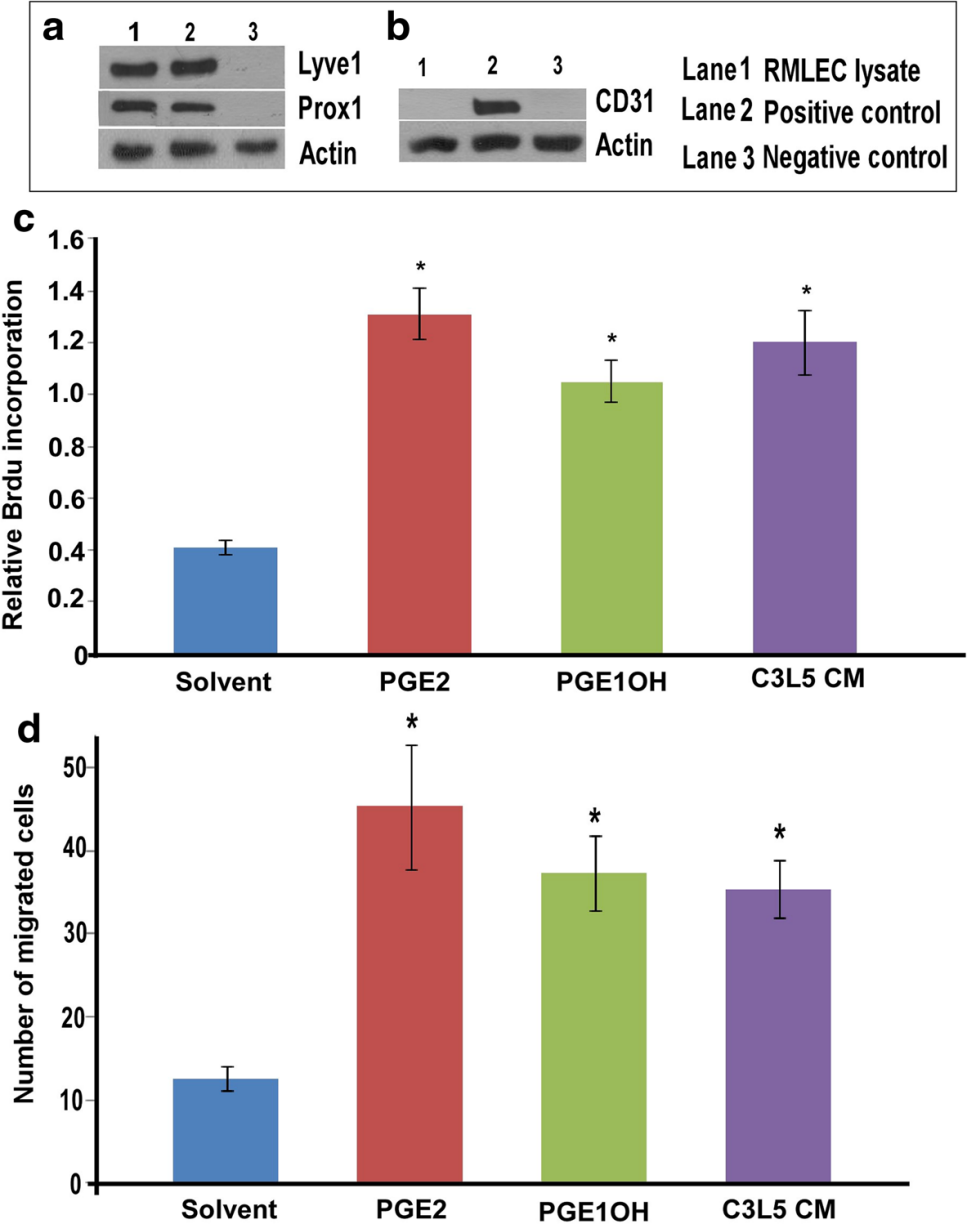
Effects of EP4 ligands and C3L5-CM on proliferation and migration of RMLEC. Western blots for **a** Lyve-1, Prox-1 and **b** CD-31 were performed with RMLEC lysate. For both Lyve-1 and Prox-1, mouse lung extract and MCF-7 cell lysate were used as respective + ve and –ve controls. For CD-31, HUVEC and HEK-293 cell lysates were used as respective + ve and –ve controls. **c** proliferation (BrdU incorporation) and **d** trans-well migration assays were performed to assess the effects of PGE2, PGE1OH and C3L5-CM on RMLEC. Error bars represent mean ± SEM of three different experiments. (*) represents *p* < 0.05

The RMLECs were tested for the expression of the lymphatic markers Lyve-1, prox-1 and angiogenesis marker CD31 by western blot. The cells were positive for Lyve-1, prox-1 (Fig. 1a), and negative for CD-31 (Fig. 1b). These findings validated the data reported by the originator of the cell line [30]. They also expressed all EP receptors (not shown).

### The effects of PGE2, EP4 agonist and C3L5-CM on proliferation and migration of RMLECs

Lymphangiogeneisis *in vivo* involves proliferation, migration and organization of LEC into complex tubular structures that can crudely be recapitulated in vitro in individual assays. Based on preliminary experiments showing that both PGE2 and PGE1OH stimulated these events equally at 10 μM and 20 μM concentrations, we used the lower concentrations in our assays except with EP4 KD cells (shown later, in which case we used the higher concentration). Presence of non-selective EP ligand PGE2 (10μM), EP4 agonist PGE1-OH (10μM) and 24h C3L5-CM significantly stimulated the proliferation (Brdu uptake for 24 h) (Fig. 1c) and migration (24h in trans-well) (Fig. 1d) of RMLEC in media containing 2% charcoal-stripped FBS.

### Tube formation by RMLEC under various treatment conditions: Effects of PGE2, EP4 agonist, C3L5-CM, COX1/ COX-2 inhibitor and EP4 antagonist

**Fig. 2:**
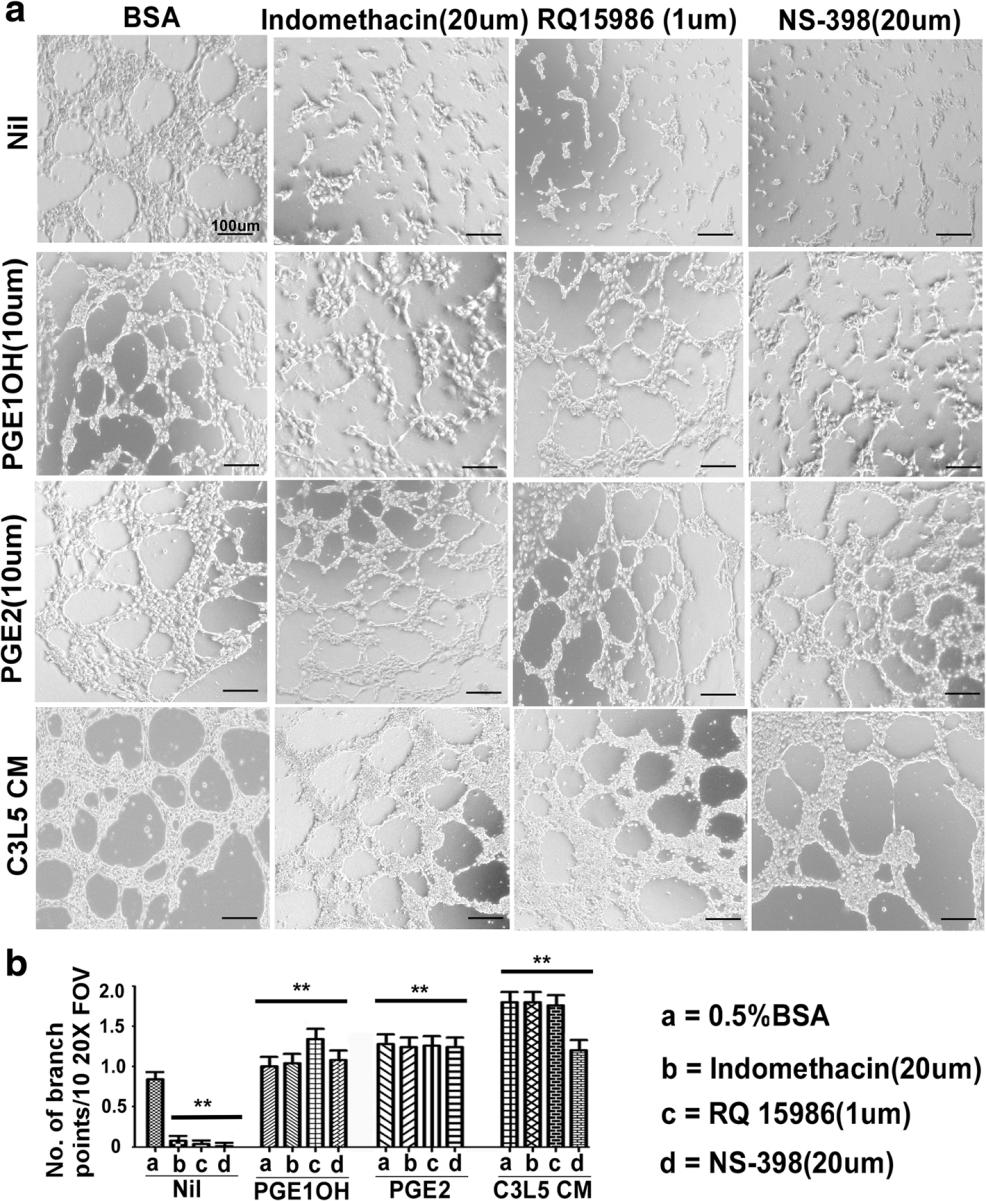
Representative photo-micrographs of RMLEC tubular network formation in response to different agents. **a** Tube formation were measured on GFR-Matrigel at 30 h in the presence of BSA(control), COX-1/2 inhibitor indomethacin (20μM), COX-2 selective inhibitor NS-398 (20μM), EP4 antagonist RQ-15986 (1μM), PGE2 (10μM), EP4 agonist PGE1-OH (10μM), and 24h C3L5-CM. Bars equal 100 μm. **b** Quantification of the number of branching points in the captured area representing 10 different fields of view, (FOV) for each experimental condition. Each bar graph represents each experimental condition. Error bars represent mean ± SEM of three different experiments. (**) represents *p* < 0.001

RMLECs cultured overnight in advanced-DMEM with 2% FBS were washed and seeded on GFR Matrigel-coated plates (40,000 cells per well), and tube formation examined at 24–96 h. Under native conditions tube formation was sparse at 12 h, but evident at 24 h. This was further stimulated by EP4 agonist PGE1OH (10μM) or PGE2 (10μM) or cell-free C3L5-CM, whereas non selective COX-1/COX-2 inhibitor Indomethacin (20μM), selective COX-2 inhibitor NS-398 (20μM) and selective EP4 antagonist RQ15986 (1μM) abrogated the native tube formation (Fig. 2a). C3L5-CM collected from C3L5 cells pre-treated for 24h with COX-2 inhibitor NS-398 (20μM) had no stimulatory activity (not shown). Since C3L5 cells secreted VEGF-C/D as well as PGE2 which are abrogated with COX-2 inhibitor and C3L5-CM collected after pretreatment of C3L5 cells may have unspent COX-2 inhibitor, we could not definitely identify the reasons for the loss of stimulation. This prompted us to interrogate the roles of exogenous PGE2 or EP4 agonist on tubulogenesis. Quantification of tubulogenesis given as the numbers of branching points under each condition is presented in (Fig. 2b). These results indicated that Matrigel induced tube formation was dependent on COX-2 and EP4 activity of the LEC. This is consistent with our findings that LEC expressed very little COX-2 in monolayer cultures but both COX-2 and EP4 were upregulated in the presence of Matrigel (data not shown). Additional presence of PGE1OH (10μM) or PGE2 (10μM) or C3L5-CM to the RMLECs restored tube formation in the presence of the above inhibitors or antagonists (Fig. 2a). That EP4 agonist could restore tube formation in the presence of the antagonist suggested that the antagonist did not completely block all EP4 receptors. This led to the subsequent experiments exploring the EP4 dependence of tubulogenesis as presented later using EP4 KD RMLEC.

### Tube formation by human dermal LEC (HMVEC-dly) under various treatment conditions

**Fig. 3:**
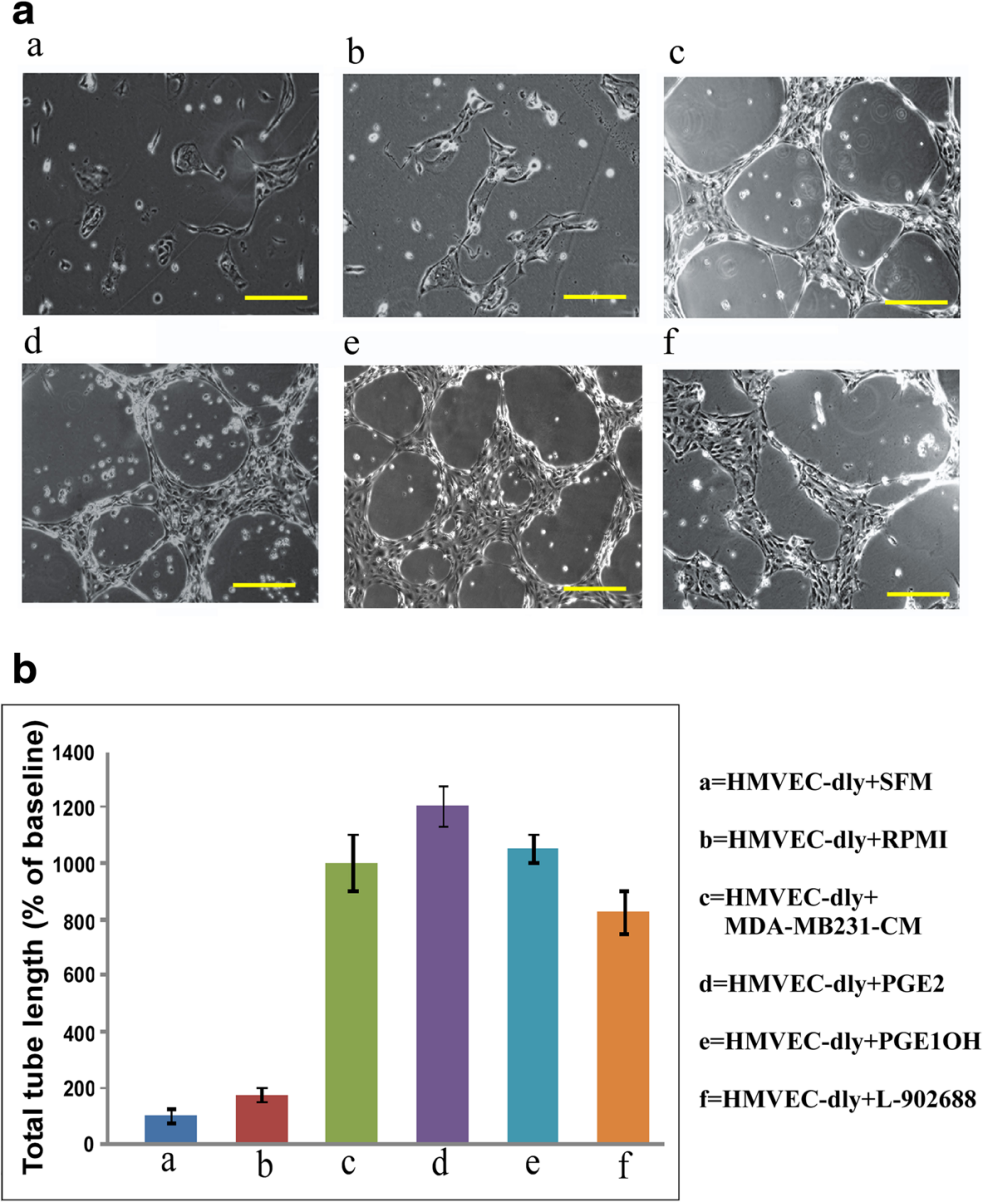
Representative micrographs of HMVEC-dly tubular network formation in response to different agents. **a** Tube formation were measured on GFR-Matrigel at 24 h in the presence of SFM or RPMI medium (control), MDA-MB-231 CM, PGE2 (1μM), EP4 agonists PGE1OH (1μM), and L-902688. Bars equal 100 μm. **b** Quantification of the total tube length in the captured area at 10 different field of view, (FOV) for each experimental condition as determined by ImageJ. Each bar graph represents each experimental condition. Error bar represents mean ± SEM of three different experiments. (**) represents *p* < 0.001. Tubulogenesis quantitated by innumeration of branching points gave identical results (not shown)

We tested whether some of the results noted above in RMLEC were reproducible with human LEC (HMVEC-dLyAd) in tubulogenesis assays. In this case we used cell free conditioned medium from a high COX-2 expressing MDA-MB-231 cells [26]. Representative images and quantification at 24 h are presented respectively in Fig. 3a and b. Addition of MDA-MB-231-CM, PGE2 and two EP4 agonists (PGE1OH and L-902688) all markedly stimulated tubulogenesis (*p* < 0.001), simulating the results noted above with RMLEC.

### Migration and tube formation by EP4 KD RMLEC under different treatment conditions

**Fig. 4:**
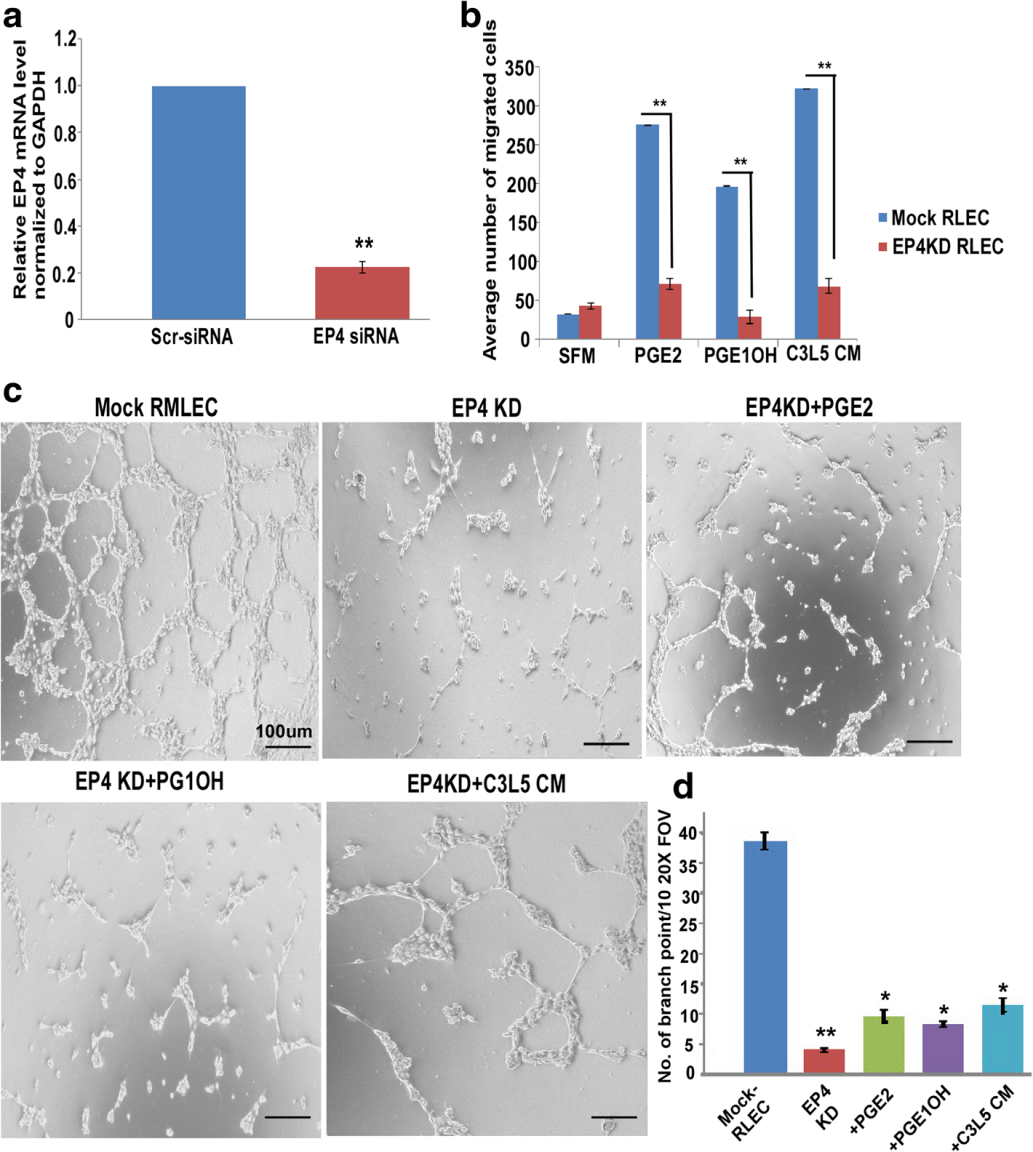
Effects of EP4 knock-down on migration and tube formation by RMLEC. **a** Real-time qPCR of EP4 mRNA expression (normalized to GAPDH) in EP4 knock-down RMLEC. **b** Cellular migration of EP4 KD RMLEC in response to PGE2 (20μM), EP4 agonist PGE1OH (20μM), and 24h C3L5-CM. **c** Tube formation by EP4 KD RMLEC on GFR-Matrigel at 24 h in the presence of the stimulating agents at same concentrations used for migration assay. Bars equal 100 μm. **d** Quantification of the number of branching points in the captured area at 10 different FOV for each experimental condition as determined by imageJ. Error bar represents mean ± SEM of three different experiments. (*) and (**) represent *p* < 0.05 and *p* < 0.001 respectively

EP4 shRNA transfected RMLECs exhibited about 80% EP4 knockdown (KD) as demonstrated by qPCR relative to mock (scrambled shRNA)-transfected (control) cells (Fig. 4a). EP4 KD cells exhibited 98–99% viability, as assessed by trypan blue exclusion. Both mock and EP4 KD cells were subjected to migration (24 h) and tube formation (30 h) assays in the presence or absence of PGE2 (20μM), PGE1OH (20μM) or C3L5-CM. While all these agents significantly stimulated migration in control cells (Mock) none of these agents could significantly restore migration of EP4 KD cells to control levels (Fig. 4b). The number of migrant cells were not significantly different from those in the absence of these agents. Tube formation in control cells (shown in Fig. 4c) was similar to those of untransfected cells (not shown). EP4 KD cells showed significant loss of ability for tubulogenesis as compared to control cells (Fig. 4c). Addition of non-selective EP ligand PGE2 (10μM), selective EP4 agonist PGE1OH (10μM) or C3L5-CM to the EP4 KD cells failed to significantly restore tube formation (morphology in Fig. 4c, quantification in Fig. 4d). These results establish that migration and tubulogenesis by RMLEC were EP4 dependent.

### Effect of the C3L5-CM and EP ligands on moleluclar profile of RMLEC

**Fig. 5:**
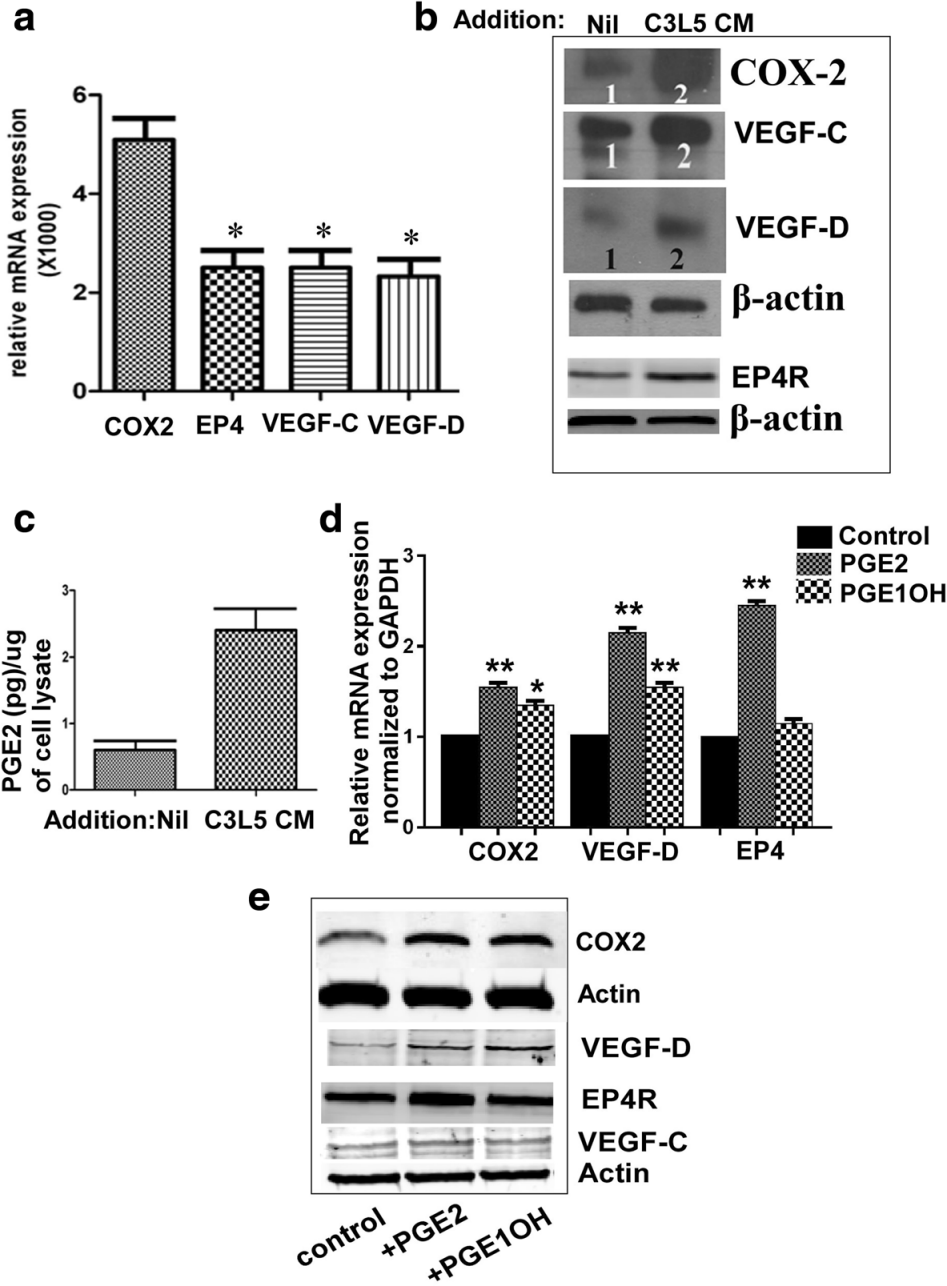
Effects of C3L5-CM, PGE2 and PGE1OH on molecular profile of RMLEC. **a** Relative mRNA level of COX-2, EP4, VEGF-C, VEGF-D in RMLEC treated with C3L5 -CM for 24 h compared to control. **b** Western blots with cytoplasmic extracts obtained from above treated cells showing the expression of EP4, COX-2, VEGF-C, VEGF-D at the protein level. **c** Cytoplasmic PGE2 level (measured by ELISA) in C3L5-CM treated RMLEC compared to control. **d** Relative COX-2, VEGF-D and EP4 mRNA expression (normalized to GAPDH) in untreated (control) or PGE2 and PGE1OH treated RMLEC. **e** Western blots of COX-2, VEGF-D and EP4 protein in untreated or PGE2 and PGE1OH treated RMLEC. Error bar indicates mean ± SEM. (*) and (**) indicates p values < 0.05 and <0.001 respectively

Since in our preliminary differential gene array experiment (data not shown) we observed significant upregulation of a number of molecules including COX-2, VEGF-C, VEGF-D and EP4 in C3L5 CM treated RMLEC, we tested the expression of these molecules by qRT-PCR and western blot. Figure 5a reveals that mRNA levels of the above molecules were significantly upregulated by C3L5-CM. Figure 5b shows upregulation of COX2, VEGF-C, VEGF-D and EP4 also at protein levels. In support of COX2 upregulation, these cells also exhibited an increase in endogenous PGE2 production as shown with ELISA in the cell lysate (Fig. 5c). Since C3L5-CM was known to contain PGE2 in addition to other molecule such as VEGF-C and D, we could not distinguish amongst these molecules which exerted the effects. For this reason, we treated the cells directly with PGE2 and also the selective EP4 ligand PGE1OH. The results for mRNA expression are shown in Fig. 5d (qRT-PCR). Both these ligands upregulated VEGF-D and COX-2 but not VEGF-C. EP4 receptor was also upregulated only with PGE2 but not PGE1OH treatment. These data are supported by western blots (Fig. 5e).

### EP4 mediated signaling events in tubulogenesis

**Fig. 6:**
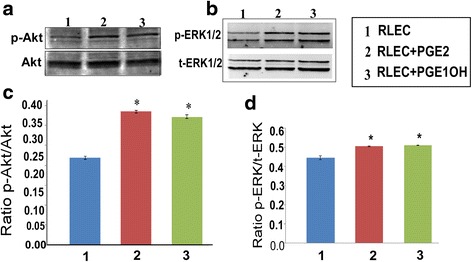
Effects of EP ligands on PI3K/Akt and Erk phosphorylation in RMLEC. Western blot analysis of (**a**). Akt and (**b**). Erk1/2 activation in RMLEC following treatment with PGE2 and PGE1OH for a period of 24 h. Stimulation of phosphorylation of Akt at Ser 473 and Erk1/2 were observed compared to control, untreated cells. Total Akt and total Erk1/2 confirmed the equivalent loading of lanes. (**c**) and (**d**). Densitometric analysis revealed that phosphorylation of both pathways was significantly induced after the treatments. (*) indicates p values < 0.05

**Fig. 7:**
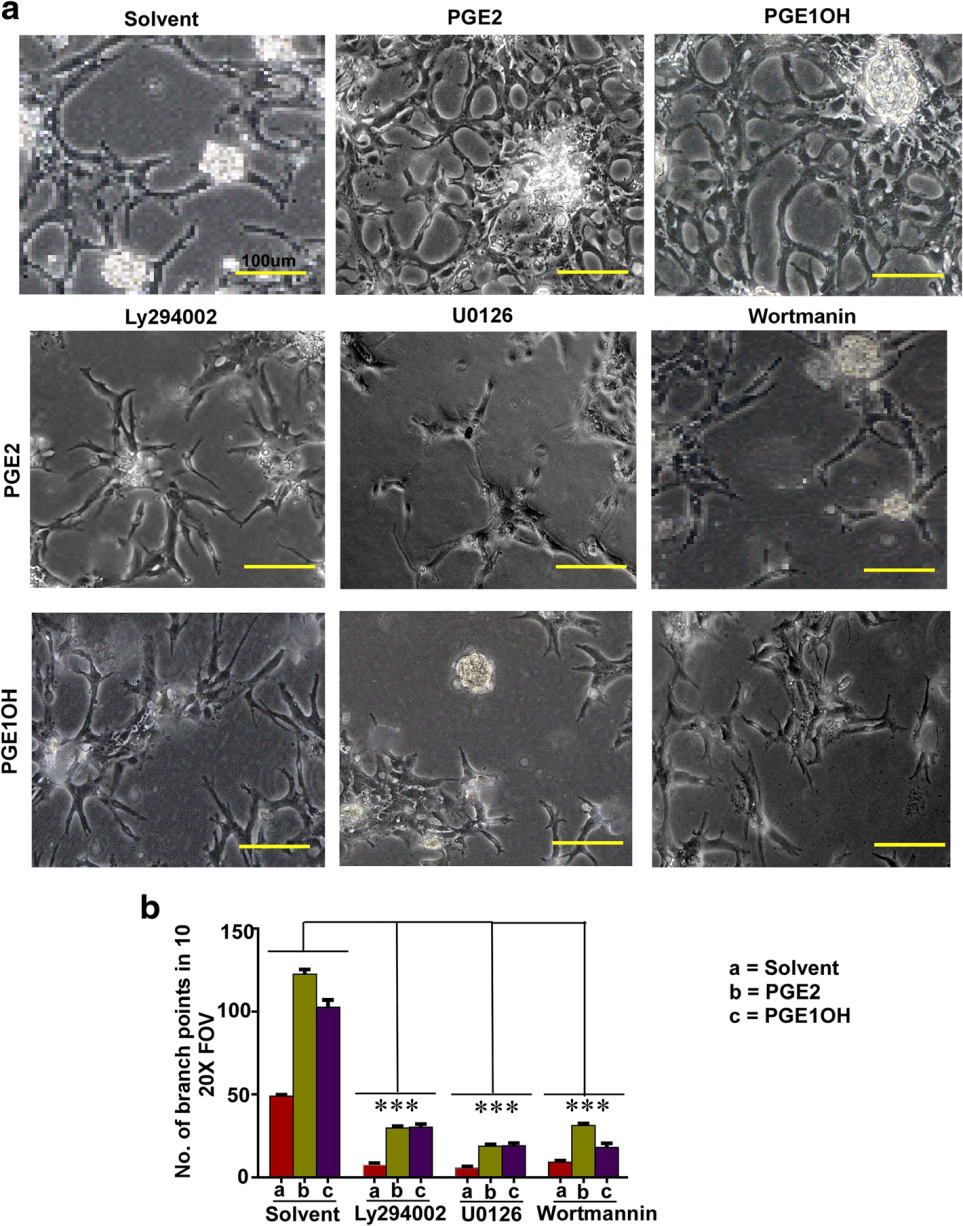
Effects of PI3K/Akt and Erk inhibitors on tube formation by RMLEC. **a** Representative photo-micrographs of RMLEC tubular network formation in response to PGE2 and PGE1OH in presence of PI3K/Akt inhibitors (Ly294002 and wortmanin) or Erk inhibitor (U0126). Bars equal 100 μm. **b** Quantification of branch points of tubular structures corresponding to (**a**) as determined by ImageJ. (***) represents *p* = 0.0001

While both EP2 and EP4 receptors share signaling by cAMP pathway, EP4 also known to utilize non-canonical PI3K/Akt and Erk pathways [33]. For this reason we examined Akt and Erk phosphorylation in RMLEC following both PGE2 and EP4 agonist (PGE1OH) treatment. Both Akt and Erk1/2 phosphorylation were stimulated at 24 h (relative to total Akt or total Erk) with both ligands (Fig. 6a, b). Whether these pathways were essential for agonist mediated tubulogenesis was examined in RMLEC treated with PI3K/Akt inhibitor (LY294002, wortmannin) and Erk inhibitor (U0126). Both pathway inhibitors effectively blocked tubulogenesis in the presence of the above ligands indicating the requirement of both pathways in EP4 mediated tubulogenesis (Fig. 7a, b). However these inhibitors also significantly reduced native tubulogenisis on Matrigel indicating the obligatory need of both pathways for Matrigel-induced tubulogenesis.

**Fig. 8:**
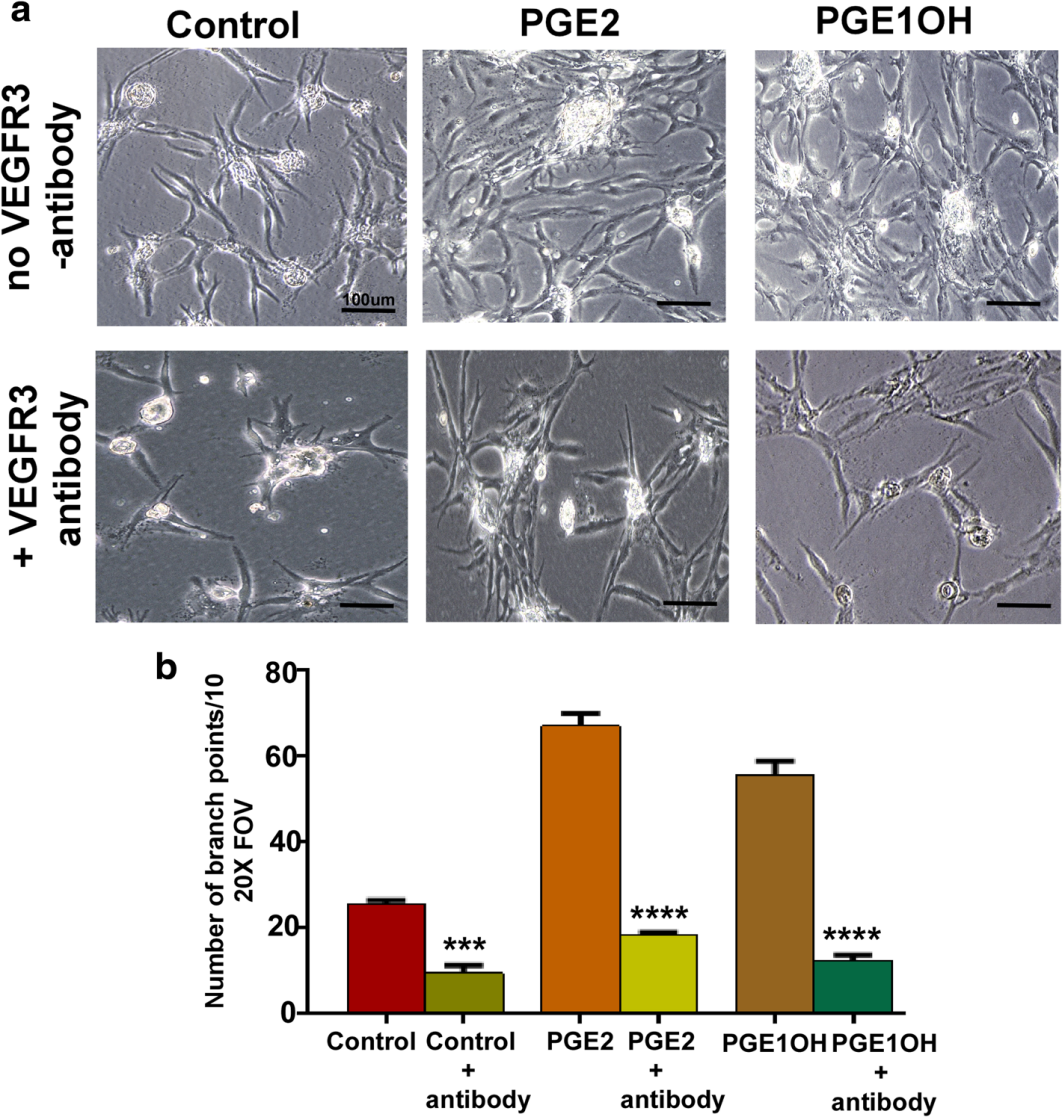
Effects of VEGFR-3 neutralizing antibody on tube formation by RMLEC. **a** Representative images of RMLEC tube formation in response to treatment with VEGFR-3 neutralizing antibody (5μg/ml). Presence of PGE2 or PGE1OH could not reverse the VEGFR-3 antibody mediated inhibition of tube formation. Bars equal 100 μm. **b** Quantification of branch points of tubular structures corresponding to (**a**) as determined by ImageJ. Data are presented as mean ± SEM. (***) represents *p* = 0.0001; (****) represents *p* < 0.0001

Since VEGF-D was upregulated in LEC following both PGE2 and PGE1OH treatment (Fig. 5d, e), it was likely that EP4 mediated tubulogenesis noted above was an indirect result of VEGF-D production and its binding to its receptor VEGFR-3. To test this hypothesis, we examined EP4 agonist mediated tubulogenesis in the presence of VEGFR-3 blocking antibody. Presence of the antibody significantly blocked native tube formation and tubulogenesis induced by PGE2/ PGE1OH (Fig. 8), suggesting the obligatory role of VEGFR-3 in tubulogenesis in RMLEC.

### In vivo angiogenesis and lymphangiogenesis assay

**Fig. 9:**
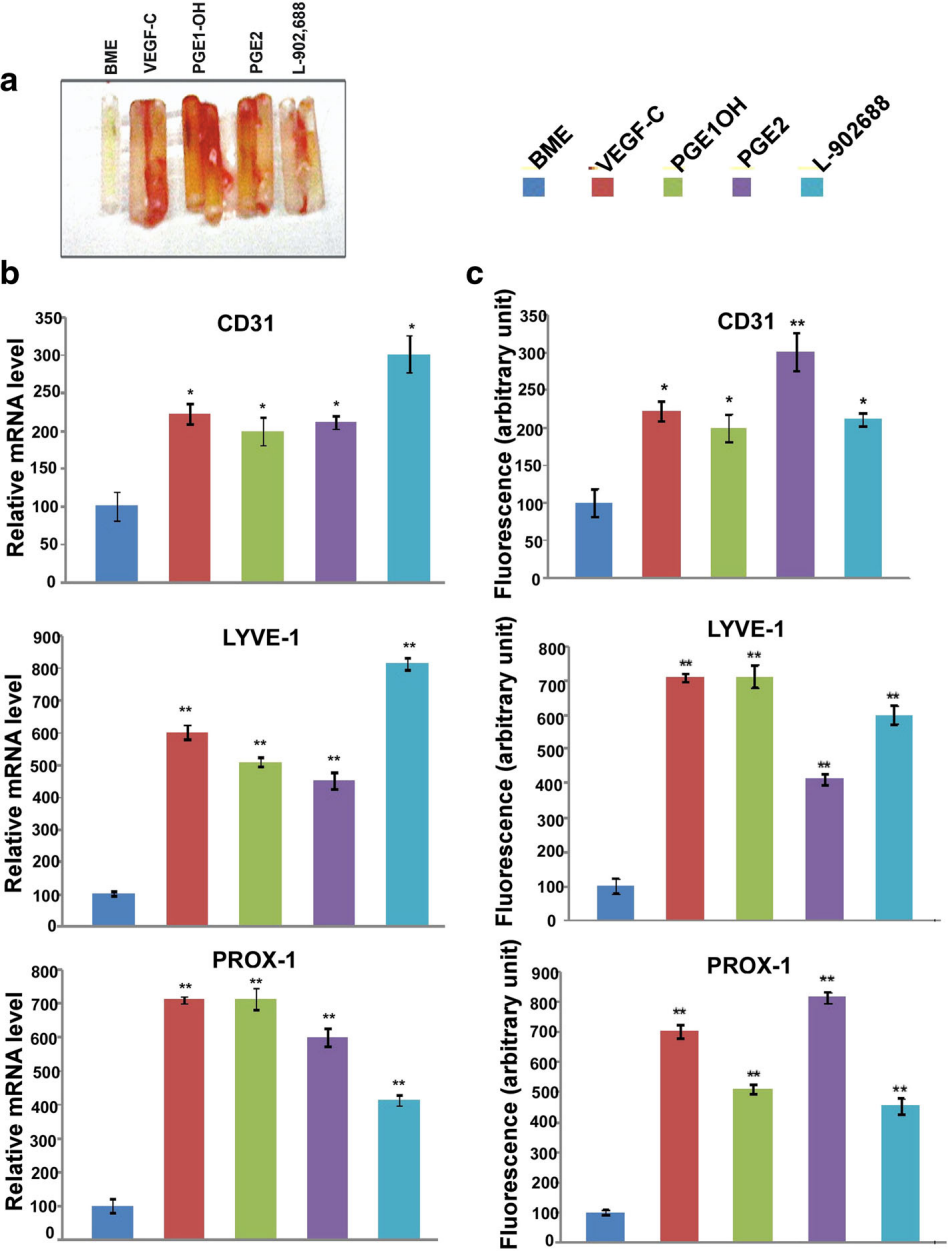
Expression of angiogenesis and lymphangiogenesis markers in angioreactors obtained at DIVLA **a**. Macroscopic images of angioreactors collected with surrounding tissues. **b** Quantitative real-time PCR analysis of Lyve1 Prox-1 and CD31 mRNA expression in the cellular contents of angioreactors. Expression levels are normalized to actin. **c** relative fluorescence analysis of Lyve1, Prox1, and CD31 proteins as expressed by mouse lymphatic and blood vascular endothelial cells recruited into the angioreactors. Data are presented as mean ± SEM. (*) indicates significant difference *p* < 0.05 and (**) indicates *p* < 0.001

**Fig. 10:**
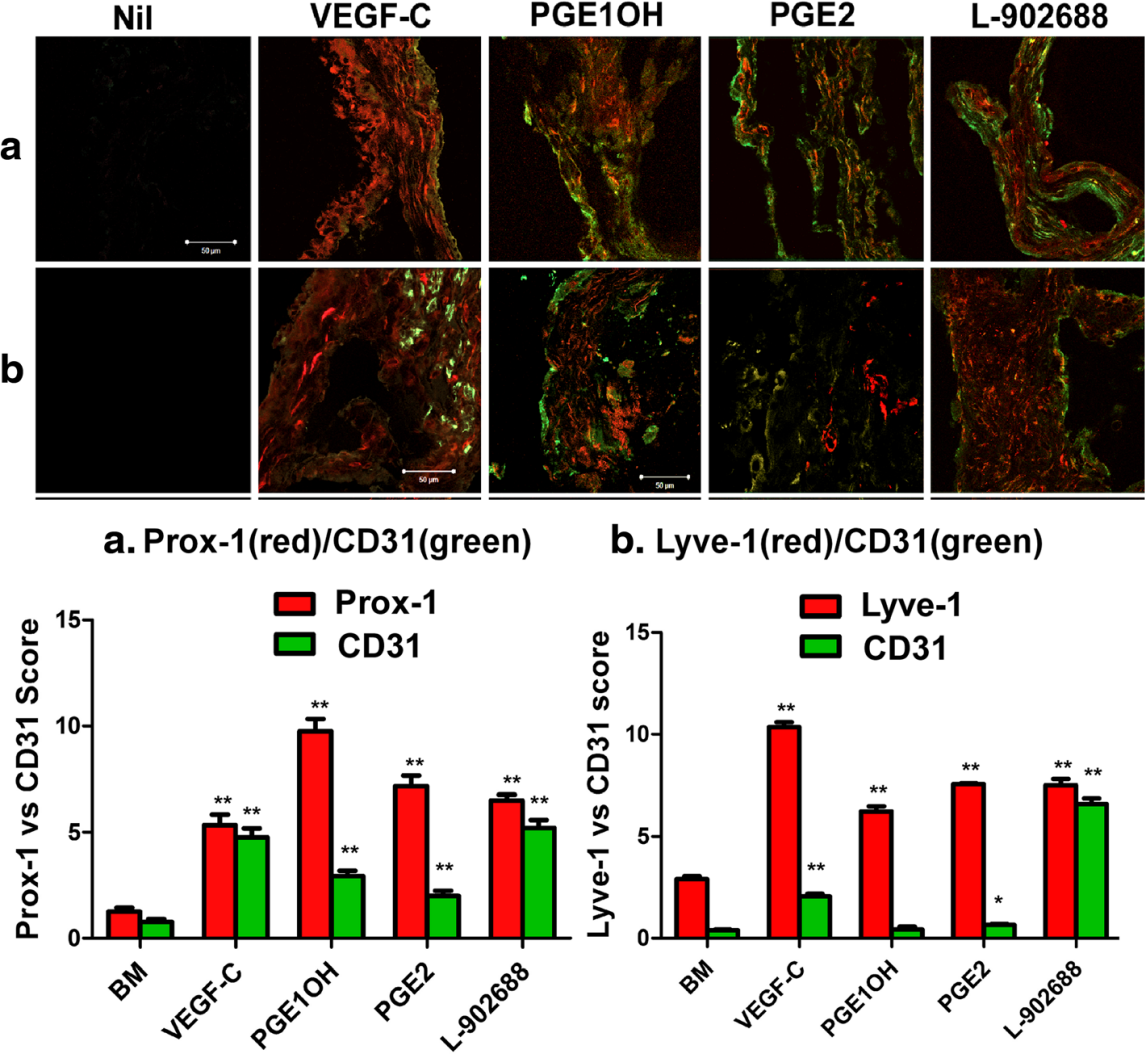
Immuno-histological quantification of lymphatics and vascular endothelial cells in angioreactor contents based on double labeling. (*Upper panel*). Immunofluorescence localization of CD31 (*green*), Lyve1 (*red*), and Prox1 (*red*) in serial sections of angioreactors containing BME alone or BME + VEGF-C (5ng/200μl), BME + PGE1OH (10μM), BME + PGE2 (10μM), BME + L-902688(10μM). Images were captured using the confocal microscope. (*lower panel*). “Hot spot” scores for CD31-Lyve1 and CD31-Prox1 were calculated by means of Image J (40× magnification). Data are presented as mean of “hot spot” ± SEM. Scale bar equals 50 μm. (*) indicates significant difference *p* < 0.05 and (**) indicates *p* < 0.001 relative to BME (control) for each marker

Directed in-vivo angiogenesis and lymphangiogenesis assays in mice [29] was performed by implanting the angioreactors with VEGF-C, PGE2, and EP4 agonists PGE1OH or L-902,688 mixed in BME. After ten days, the angioreactors were removed to visualize their gross appearances (Fig. 9a) which revealed red colors (crude evidence of angiogenesis) variably in all ligand containing angioreactors. Their contents were used to quantify the lymphatic and blood vascular in-growth as given by the expression of lymphatic and vascular endothelial cell markers using qPCR (of mRNA) (Fig. 9b) and spectrofluorometry (of proteins) (Fig. 9c). VEGF-C (used as the internal positive control), PGE2 and both EP4 agonists increased the protein and mRNA expression levels of Prox-1 and Lyve-1. Histological examination of angioreactors were carried out by dual immune-staining of cryo-sections for Lyve-1, or Prox-1, and CD-31. Inclusion of VEGF-C, PGE2 (10μM) or EP4 agonists PGE1OH (10μM) or L-902688(10μM) in the angioreactors markedly increased the incidence of in-growing lymphatics (stained for Lyve-1or Prox-1) and to a smaller extent blood vessels (stained for CD31) (Fig. 10) into the angioreactors.

## Discussion

Inflammation is believed to play an important role during all aspects of tumor development and tumor progression [34]. It is also an important promoter of lymphangiogenesis. There are possibly many common inflammation-associated mediators participating in both events. Of these, the role of tumor or host derived PGE2 in lymphangiogenesis has not been well-defined. Among the multiple tumor cell products stimulating breast cancer associated lymphangiogenesis, we have earlier reported the roles of VEGF-C and VEGF-D produced by COX-2 expressing breast cancer cells in the human [26] as well as in the mouse [27]. Furthermore, VEGF-C and –D production by tumor infiltrating macrophages also contributed to lymphangiogenesis in our murine breast cancer model [25]. In addition COX-2 expression by breast cancer cells can upregulate the expression of the chemokine receptor CCR7 via activation of EP2/EP4 receptors [35]. We have shown that a molecular cross-talk between breast cancer cells and LEC via the CCL21/CCR7 chemokine/ chemokine receptor axis plays a major role in breast cancer associated lymphangiogenesis [15]. In the present study we used a rat mesenteric lymphatic endothelial cell line which expressed the lymphatic markers Lyve-1 and Prox-1 but not the angiogenesis marker CD31 and a COX-2 expressing murine breast cancer cell line C3L5 to study the roles of soluble tumor cell products on lymphangiogenesis. Tube formation by the LEC *in vitro* is a complex morphological event that only partially recapitulates lymphangiogenesis *in vivo*, requiring cell migration, alignment and formation of three-dimensional structures that mimics lymphatic capillaries. In spite of limitations, it is a valuable *in vitro* model to dissect the mechanisms without the influence of confounding factors that can modulate lymphangiogenesis *in vivo* in a positive or a negative manner [28]. We had earlier shown that C3L5 cells secrete PGE2 and lympangiogenic factors VEGF-C and VEGF-D [27]. To dissect the roles of tumor-derived PGE2 and EP4 receptors on the LEC in the lymphangiogenic events, we measured the effects of C3L5- CM, exogenous PGE2 and EP4 agonists on LEC proliferation, migration and tubulogenesis, showing a stimulation of all the three events with all of the above agents. Conversely, natural tube formation by the LEC on GFR-Matrigel was abrogated in presence of COX-1/2 inhibitor Indomethacin, COX-2 inhibitor NS-398 and EP4 antagonist RQ15986, indicating that Matrigel induced tubulogenesis was dependent on COX-2 and EP4 activity of the LEC. Tubulogenesis was largely restored by the additional presence of PGE2 or EP4 agonist or C3L5-CM. These findings taken together reinforce the stimulatory roles of tumor-derived PGE2 in breast cancer associated lymphangiognesis by activation of EP4 expressed by the LEC. We have replicated some of these findings using human dermal LEC (HMVEC-dly) and a COX-2 expressing human breast cancer cell line MDA-MB-231. These in vitro results using the LECs and breast cancer cells suggest that PGE2 is another tumor-derived molecule that can directly stimulate lymphangiogenesis by binding to EP4 on the LEC.

C3L5-CM contains multiple lymphangiogenic molecules including PGE2, VEGF-C and VEGF-D [27] that can directly act on the LEC. Similarly, MDA-MB-231-CM contains at least two classes of stimulatory molecules namely VEGF-C and PGE2, both produced in a COX-2 dependent manner [26]. While VEGF-C and VEGF-D can stimulate LEC growth and tubulogenesis by binding to VEGFR-3 [28], PGE2 could promote these events by activating EP4. The fact that shRNA-mediated EP4 silencing of RMLEC made them refractory to the stimulation by the C3L5-CM or PGE2/EP4 agonist (Fig. 4) reinforced the obligatory role of EP4 in lymphangiogenesis.

**Fig. 11:**
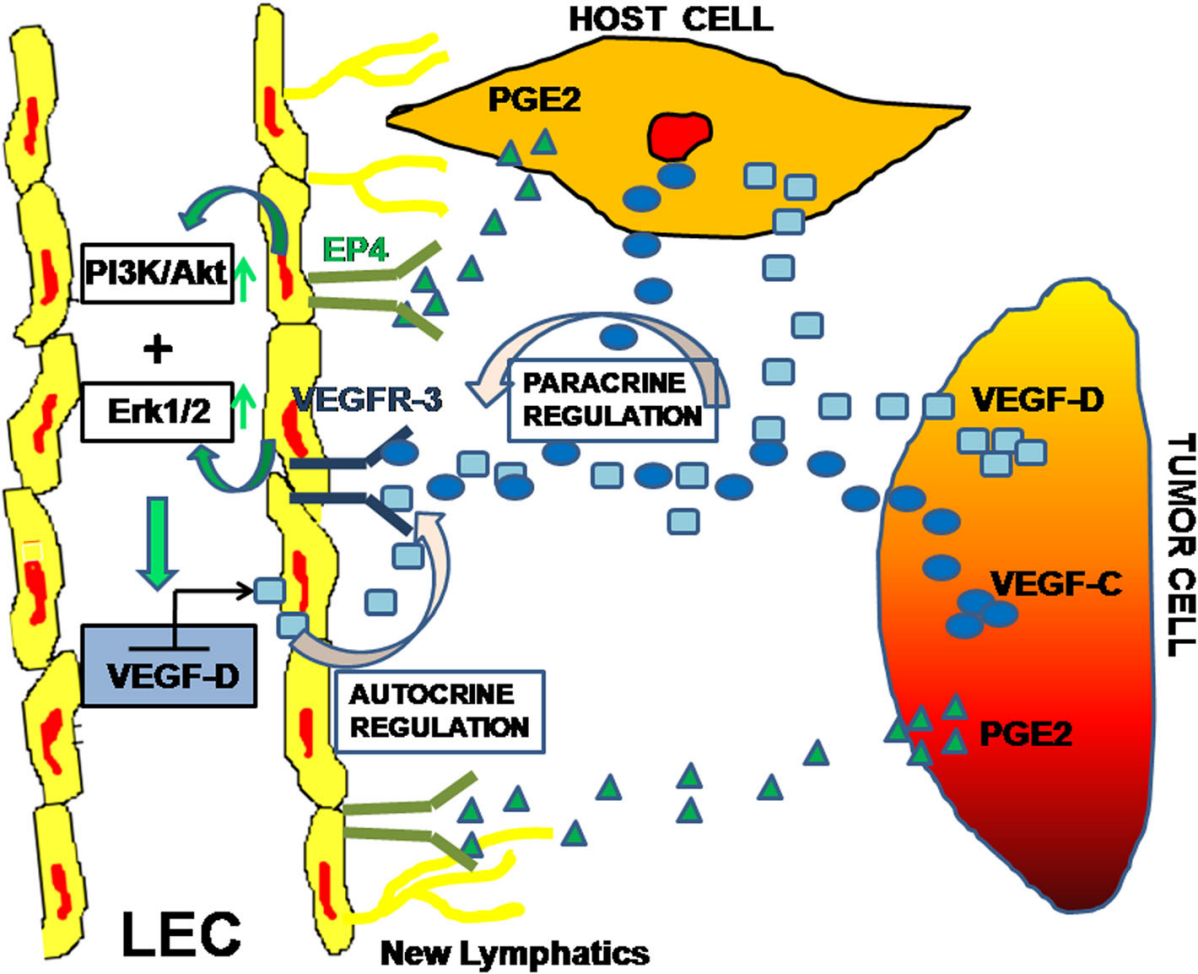
Proposed roles of PGE2-EP4 in breast cancer associated lymphangiogenesis. Molecular cross-talk amongst tumor cells, host cells (macrophages) and LECs: Both tumor and host cells produce PGE2 and VEGF-C/D. PGE2 binding to EP4 receptors on LEC directly promote lymphangiogenesis (via proliferation migration and tubulogenesis) following activation of PI3K/Akt and Erk signaling. EP4 activation also upregulates VEGF-D production by the LEC. VEGF-C/D derived from multiple sources (autocrine and paracrine) stimulates sprouting of new lymphatic vessels by binding to VEGFR-3 on LEC

PGE2 in the tumor microenvirnment *in vivo* can be a product of tumor cells or host cells such as macrophages. Indeed this is also true for the lymphangiogenic factors VEGF-C or VEGF-D [28]. EP4 activation stimulates cAMP/PKA pathway shared by EP2, and also stimulates non-canonical PI3K/Akt and Erk1/2 pathways not shared by EP2 [33]. We show here that PGE2 or EP4 agonist mediated stimulation of tubulogenesis in the RMLEC was dependent on both PI3K/AkT and Erk pathways. Unexpectedly, C3L5-CM also stimulated the production of PGE2, VEGF-C, VEGF-D by the RMLEC and uptregulated COX2 and EP4 in RMLEC (Fig. 5). Similarly PGE2 or EP4 agonist upregulated VEGF-D in RMLEC. Thus VEGF-C and VEGF-D in turn could lead to an autocrine growth stimulation by activation of VEGFR-3 on the LEC. This contention was fully validated by the fact that EP4 agonist mediated tubulogenesis was abrogated in presence of VEGFR-3 blocking antibody. These results lead to the proposal that there is a molecular cross talk between breast cancer cells or host macrophages and LEC involving EP4/PGE2 axis and VEGF-C or D/VEGFR-3 axis (schema presented in Fig. 11).

In the present study we validated the roles of PGE2 and EP4 in lymphangiogenesis *in vitro* and *in vivo* studies in nude mice using directed in vivo lymphangiogenesis assay (DIVLA) previously developed in our lab [29]. Inclusion of PGE2 and two EP4 agonists in the angioreactors significantly increased lymphangiogenesis and to a minor extent angiogenesis, identified by three approaches: expression of lymphatic endothelial marker molecules Prox-1 and Lyve-1 and vascular endothelial marker molecule CD31 at the mRNA and protein levels; and a dual immunostaining of lymphatics for Lyve-1 or prox-1 and blood vessels for CD31. Thus we confirmed the lymphangiogenic as well as angiogenic capacity of PGE2 and EP4a in vivo. Our results support a recent report that PGE2 promotes angiogenesis through EP4 activation [36].

All the above results cumulatively demonstrate the roles of tumor as well as host-derived PGE2 in inducing lymphangiogenesis, at least in part, by activating EP4 on lymphatic endothelial cells. Since EP2 and EP4 share cAMP signaling pathways, we believe that EP2 is another player. Absence of highly selective EP2 agonists has precluded testing whether EP4 is more lymphangiogenic than EP2.

We found in multiple studies that EP4 on breast cancer cells accounts for numerous COX-2 mediated mechanisms in breast cancer progression: increased migration and invasion [22–24], VEGF-C/D upregulation [26, 27] promotion of tumor-associated angiogenesis and lymphangiogenesis in vivo [27] and finally, induction of stem-like cells [37, 38]. Using the COX-2 expressing murine C3L5 breast cancer model, we found that an EP4 antagonist at nontoxic doses abrogated tumor growth, tumor associated angiogenesis and lymphangiogenesis, and metastasis to the lymph nodes and the lungs, and an abrogation of stem-like cell functions in *vitro* and in *vivo* [25, 27]. Present results reveal that EP4 is also a common target on the LEC for blocking direct effects of tumor or host derived PGE2 on lymphangiogenesis. Since long term use of COX-2 inhibitors are reported to increase thrombo-embolic side effects in patients [39–41], there is a continuous search for drugs targeting molecules downstream of COX-2, that may spare cardio-protective prostacyclins such as PGI2 [42]. We suggest that EP4 antagonists may satisfy this requirement and can be utilized in the intervention of lymphatic metastasis in breast cancer.

## Conclusions

Present results add a new dimension in translational breast cancer research. EP4 is a receptor shared by tumor cells [26, 27] as well as host cells e.g., NK cells [43], macrophages [25] and LEC (current study), responsible for multiple COX-2 mediated cellular processes responsible for breast cancer progression and metastasis (Schema presented in Fig. 11). Relative physiological redundancy of EP4 receptor due to sharing many of its functions by EP2 via cAMP pathway makes it a logical therapeutic target in breast cancer. In a phase1/II study of arthritis patients, EP4 antagonists at therapeutic doses have shown high tolerability (Dr Yukinori Take, and Dr. Akihiro Furuta, Ask/At, Japan, personal communication) deserving clinical testing as an adjuvant in breast cancer.

## Abbreviations

CCL21/6Ckine: C-C chemokine ligand 21
CCR7: C-C chemokine receptor 7
CD31: Cluster of differentiation 31
COX-2: Cyclooxygenase 2
DAPI: 4′, 6-diamidino-2-phenylindole
DIVLA: Directed in vivo lymphangiogenic assay
DNA: Deoxyribonucleic acid
ELISA: Enzyme-linked immunosorbent assay
EP: Prostanoid receptor
ERK1/2: Extracellular regulated kinase 1/2
GAPDH: Glyceraldehyde 3-phosphate dehydrogenase
HMVEC-dLyAd: Primary adult human dermal lymphatic microvascular endothelial cells
Lyve-1: Lymphatic vessel endothelial receptor-1
LECs: Lymphatic endothelial cells
PI3K: Phosphatidylinositol 3-kinase
Prox-1: Prospero homebox protein-1
AKT/PKB: Protein kinase B
PGE2: Prostaglandin E2
RMLEC: Rat mesenteric lymphatic endothelial cells
RT-PCR: Reverse transcriptase polymerase chain reaction
SLC: Stem like cell
VEGF: Vascular endothelial growth factor
VEGFR-3: Vascular endothelial growth factor receptor-3
